# International Competencies of Advanced Practice Nurses in Critical Care: An Integrative Review

**DOI:** 10.1111/jan.70179

**Published:** 2025-09-29

**Authors:** Verena‐Katrin Buchner, Kathrin Pann, Roland Essl‐Maurer, Manela Glarcher, Andre Ewers

**Affiliations:** ^1^ University Hospital Salzburg Salzburg Austria; ^2^ Paracelsus Medical University Salzburg Salzburg Austria

**Keywords:** advanced practice, clinical competence, critical care nursing, review, scope of practice

## Abstract

**Aim:**

This review explores the roles, competencies, and scope of practice of APNs in critical care based on international literature. It also derives implications for the development of advanced nursing roles in Austria.

**Design:**

Integrative review.

**Data Sources:**

The research team conducted a systematic search of PubMed, CINAHL, and Web of Science to identify relevant peer‐reviewed publications from 2007 to 2023.

**Review Methods:**

A systematic search of electronic databases was undertaken, following Whittemore and Knafl's five‐step methodology. The included publications met the defined inclusion criteria and were appraised for quality using the Joanna Briggs Institute critical appraisal checklists. Relevant data were extracted and thematically analysed.

**Results:**

The analysis of 14 international studies revealed recurring themes related to APN core competencies and scope of practice in critical care. These were structured according to Hamric's model. However, Austria faces several challenges, including limited legal frameworks, missing educational structures, and a lack of role clarity. These factors hinder the implementation of APN roles.

**Conclusions:**

Internationally, APNs demonstrate advanced clinical skills, provide leadership in team‐based care, and integrate evidence‐based practice. These attributes enhance patient outcomes and system efficiency. In Austria, restrictive regulations, limited education, and unclear roles hinder these competencies. Reform is needed to align with international standards, and further research should explore their implementation in Austria.

**Implications:**

A gap exists between internationally demonstrated APN competencies and the current state of advanced nursing practice in Austria. This highlights the need for clearer role definitions, regulatory frameworks, and educational strategies. Addressing this gap would strengthen APN roles and improve healthcare quality. This study highlights the need to bridge this disparity.

**Reporting Method:**

This review follows the PRISMA 2020 guidelines for systematic reviews Page et al. (2021).

**Patient or Public Contribution:**

No patient or public contribution.

**Summary**

- What does this paper contribute to a wider global clinical community?
  ○ There is insufficient evidence that APNs in Austria can fully utilise the advanced clinical skills seen internationally in critical care settings, such as airway management, ventilator adjustments, and central line insertions.
  ○ There is evidence of significant barriers in critical care environments in Austria, including restrictive policies and hierarchical systems that limit APNs' ability to lead in team‐based care and the decision‐making process.
  ○ A gap is evident in the literature regarding how the adoption of internationally recognised APN competencies in critical care could enhance patient outcomes, address workforce shortages, and improve the overall quality of care in Austria.

## 1 Introduction

Globally, advanced practice nurses (APNs) have emerged as a solution for improving the shortage and misdistribution of providers and enhancing the quality and cost‐effectiveness of care. Despite the potential gains in patient outcomes, important regulatory differences, educational requirements, and practice environments remain major obstacles to overcome. The International Council of Nurses (ICN) and other international organisations are still calling for more harmonisation in the roles and recognition of APNs to be able to meet the set international health objectives, including universal health coverage (Schirmer et al. 2025; Wheeler et al. 2022).

The development of APN roles in Europe has been heterogeneous. A pan‐European survey, representing 35 national nurses associations across the continent, showed great differences in recognition, regulation, and educational pathways for APNs, and only a minority of countries had established a formal framework for these roles (de Raeve et al. 2023). Countries like the United Kingdom, Ireland, and the Netherlands have relatively advanced systems, while others, such as Germany and Austria, are still in the early stages of recognising these roles (Schirmer et al. 2025). Moreover, a lack of uniformity in the definitions and competencies of APNs across Europe complicates their integration into healthcare systems (Kaldan et al. 2019; Wheeler et al. 2022).

Advanced Practice Nurses are globally recognised to play a crucial role in critical care. Critical care settings, often referred to as intensive care units (ICUs), are highly specialised environments designed to provide continuous, comprehensive care to patients with life‐threatening conditions. APNs in these settings manage complex acute cases, perform advanced diagnostics, and contribute to care coordination, leadership, and quality improvement (Jafari Pour et al. 2024; Yamaguchi et al. 2023). With the rise of chronic and complex diseases and an aging population, APNs, including Nurse Practitioners (NPs) and Clinical Nurse Specialists (CNSs), have emerged as vital healthcare providers in both intensive and emergency care settings (Jafari Pour et al. 2024; Yamaguchi et al. 2023). However, there is considerable variation in the scope of practice, education, and regulatory frameworks across different countries, making it very challenging to standardise competencies and ensure international mobility for APNs (Hassmiller and Pulcini 2020; Wheeler et al. 2022).

According to the ICN (2020), the educational preparation for APNs requires a minimum of a Master's degree, complemented by clinical training and role‐specific competencies. Across countries, the structure, length, and regulatory oversight of APN education vary considerably. While some countries have long‐established APN programmes embedded in higher education systems, others—such as Austria—are still in the process of defining formal pathways and national standards (de Raeve et al. 2023; Glarcher and Lex 2020). This variation in academic preparation contributes to differences in role implementation, autonomy, and legal recognition.

In this review, the term Advanced Practice Nurse (APN) is used consistently to refer to nurses practicing in expanded roles that require advanced education, clinical expertise, and decision‐making competence. This includes a variety of internationally defined roles such as NPs, CNSs, and Advanced Clinical Practitioners (ACPs). Given the country‐specific variations in terminology, regulation, and role scope, all such roles are referred to as APNs in this manuscript to ensure clarity and international comparability. Although international distinctions exist between roles such as NPs and CNSs—particularly with regard to educational pathways, clinical focus, and system‐level responsibilities—this review does not analyse them separately. The decision to apply a unified term reflects the diversity and overlap of advanced roles across countries and supports a coherent synthesis of the available evidence. Role‐specific titles used in the original studies are acknowledged but not differentiated analytically.

## 2 Background

Austria faces significant challenges in developing advanced practice roles for nurses in critical care. Unlike many countries with established pathways for APNs at master level, Austria still lacks standardised education and competency frameworks in this area (Glarcher and Lex 2020). The Austrian healthcare system is highly fragmented, with primary care mainly provided by general practitioners and limited integration of nurses into advanced roles (Lidauer and Stummer 2023).

While European countries have made significant strides in expanding APN roles, Austria has focused primarily on diploma‐level qualifications until 2016, without structured pathways for specialisation in critical care (Glarcher and Lex 2020; Schirmer et al. 2025). In 2016, the Austrian nursing education system was reformed. Since then, nurses receive a bachelor's degree after completing their generalist nursing education at universities of applied sciences (Health and Nursing Act 1997d; Education Regulation 1997a; §1). As part of this reform, the previous specialisation program in critical care was integrated into the European Credit Transfer System (ECTS). Austrian nurses who intend to work in the field of critical care must undergo a 1‐year specialisation programme comprising 60 ECTS points. Upon completion of this training, they are awarded the title of “Academic Expert” in accordance with the Health and Nursing Act Special Regulation 452 as amended (Health and Nursing Act Special Regulation 452; Paracelsus Medical University 2025). Despite some achievements for nurses in general, no overall strategy exists for the further development of APN education in critical care settings up to now (Eglseer et al. 2020; Lidauer and Stummer 2023).

The lack of formalised training at the master's level to prepare nurses for advanced practice in intensive care units still presents a significant gap. While efforts to improve nursing quality are being made, they have so far not resulted in much advancement in education for advanced practice (Eglseer et al. 2020). This situation is very worrying, considering that patient care in critical care environments is becoming increasingly complex, a context in which advanced competencies are highly required (Wheeler et al. 2022; Hassmiller and Pulcini 2020).

The development of a comprehensive framework for APN education at the master's level, with a focus on critical care competencies to meet international standards and improve patient outcomes, is essential for Austria. The framework would guide advanced clinical skills, evidence‐based practice, and interprofessional collaboration, placing Austria among those countries that have successfully integrated APNs into their healthcare systems (de Raeve et al. 2023; Yamaguchi et al. 2023). Given these structural and educational gaps in Austria and the increasing international demands on critical care services, it is essential to systematically examine the current competencies and scopes of practice of APNs in this field. The following section outlines the methodology of this integrative review.

## 3 The Review

### 3.1 Aim

In this integrative review, the research team aimed to explore the current state of roles and competencies of Advanced Practice Nurses in critical care settings at an international level by addressing the following questions:

1. Which competencies for nurses with advanced practice in critical care are described in the international literature?
2. Which scope of practice for nurses with advanced practice in critical care can be identified in the international literature?

### 3.2 Design

To present the current international status of competencies and scope of practice of advanced practice nurses in critical care, an integrative review was conducted following the methodology of Whittemore and Knafl (2005), which involves five stages: (1) problem identification, (2) literature search, (3) data evaluation, (4) data analysis, and (5) presentation of findings. The integrative review was chosen as it allows for compiling a corpus of evidence from different methodologies (Whittemore and Knafl 2005). Further, it promotes rigour and reduces bias regarding analysis, synthesis, and conclusion drawing. The Preferred Reporting Items for Systematic Reviews and Meta‐Analyses (PRISMA) guidelines (Page et al. 2021) were used for guiding the reporting of this review.

### 3.3 Methods

#### 3.3.1 Literature Search

The search strategy was developed in collaboration with experts in advanced nursing practice, a university librarian, the Austrian Association of Advanced Nursing Practice (AAANP), and the Akademische Fachgesellschaft International (AFG International). The study was registered in Prospero (CRD42022351437). A comprehensive search strategy was conducted between October and November 2023, in accordance with PRISMA guidelines, including multiple databases (PubMed, CINAHL, Cochrane Library, and Web of Science), grey literature, and manual reference checks. Additionally, studies from professional societies' websites were included to ensure literature saturation. The review focused on studies involving human participants of all cultural backgrounds, published between 2007 and 2023, and limited to publications in English and German in the acute care setting (hospital/acute care/inpatient stay). Search terms were formulated using the PICo framework, search strings were constructed in a distinct manner for each database, and included phrases such as “critical care nurse,” “acute care practitioner,” “CNS,” “nurse practitioner,” “advanced nursing,” “advanced practice,” “professional competence,” “role,” “skills,” “intensive care,” and “critical care”.

#### 3.3.2 Search Outcome

The search was conducted in PubMed (*n* = 2601), CINAHL via EBSCO Host (*n* = 2864), the Cochrane Library (*n* = 132), and Web of Science (*n* = 1103). Two researchers (VB, MG) independently assessed articles for eligibility using Rayyan software (Ouzzani et al. 2016), facilitating a blinded screening process.

#### 3.3.3 Data Extraction

**FIGURE 1:**
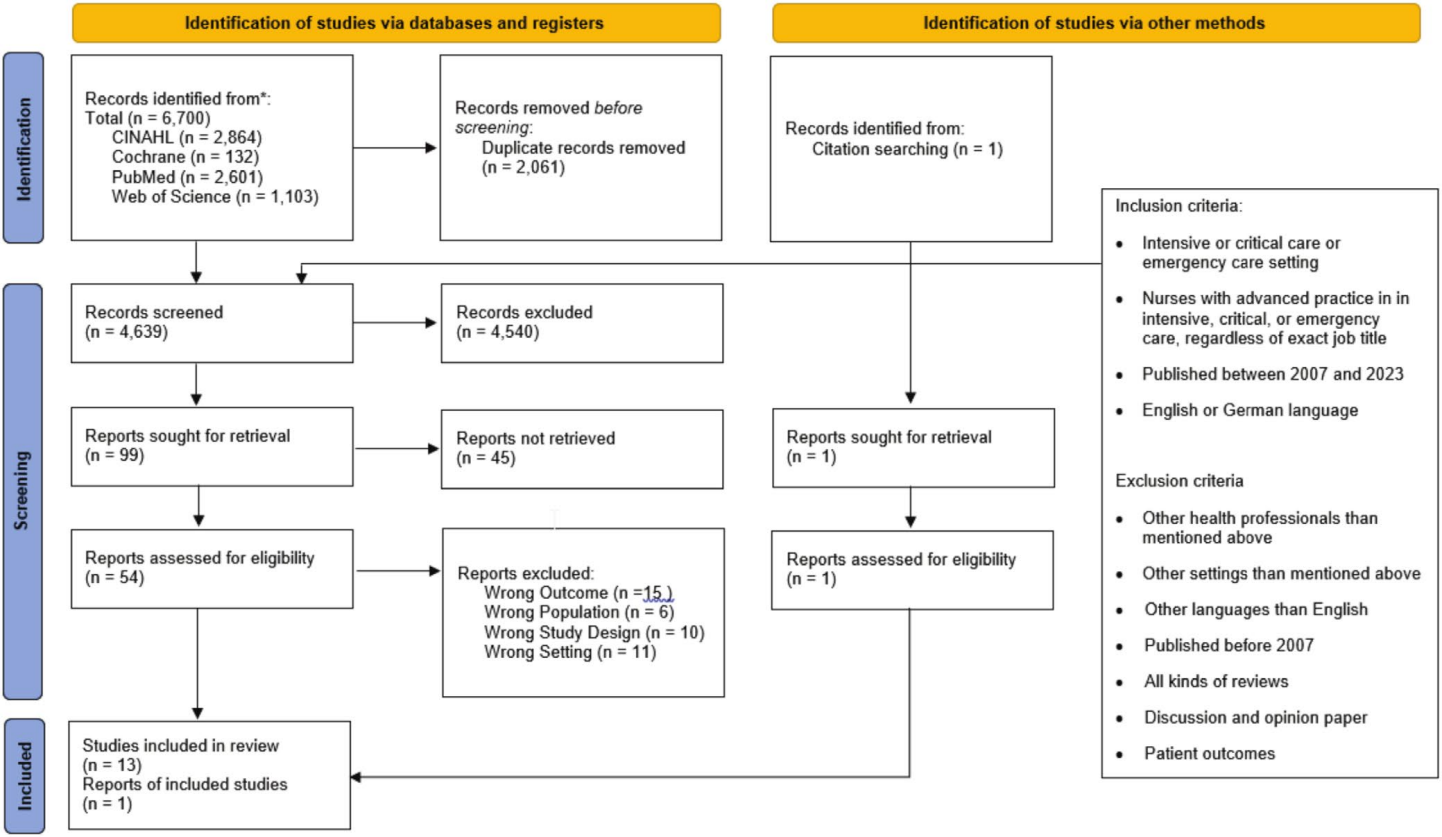
PRISMA flowchart (Page et al. 2021).

After removing 2061 duplicates, 4639 articles were initially screened by abstract review, followed by a full‐text review. Forty‐five articles were excluded due to inaccessible full texts. Discrepancies were resolved through discussion and consultation with a third expert from the research team (R.E.M.). Finally, 14 publications were included. The complete search strategy for PubMed is detailed in Supplement 1. An overview of the literature search process is presented in the PRISMA flowchart (Page et al. 2021) in Figure 1.

#### 3.3.4 Data Evaluation and Quality Appraisal

Data evaluation involved organising and assessing articles for quality and relevance using the Johns Hopkins Evidence Level and Quality Guide (Dang et al. 2021) and critical appraisal tools from the Joanna Briggs Institute (JBI), including checklists for various study designs and the Mixed‐Methods Appraisal Tool (MMAT) (Hong 2018). A summary table captured key aspects of each study: purpose, methods, sample, findings, evidence level, and quality appraisal (see Table 1). Two authors (VB, MG) independently assessed methodological quality, resolving discrepancies through discussion or consultation with a third reviewer (REM).

**TABLE 1:** Study characteristics and quality appraisal.

Nr.	References, country	Purpose	Methods	Sample	Key findings	JHNEBP	Quality appraisal
1	Crosbie et al. (2014) Scotland	Description of the Role of the Tracheostomy Clinical Nurse Specialist and recommendation of this position within the multidisciplinary team	Short communication	*n* = 1	Creation of TCNS is a benefit for both patients and team Better handling of practical and psychological issues Continuity of Care	Level V B	JBI: include (Checklist for text and opinion)
2	Crouch and Brown (2018) England	Delineation of development of UK‐wide curriculum and credentialing process for emergency care advanced clinical practitioners and reports on progress to data	Symposium	n.a.	Need for definition of advanced practice in emergency care Competency‐based approaches may lead to richer skill mixes while maintaining quality and safety EC‐ACPs are seen as part of the multi‐professional workforce with intention to complement existing medical workforce	Level IV B	JBI: include (Checklist for text and opinion)
3	Denton et al. (2022) England	Consideration of the development of airway management skills within a single advanced critical care practitioner team (scope of practice). Analysis of the safety profile in performing these aspects of critical care	Quantitative (Audit) 49‐item Google Forms	*n* = 675 ACCP intubations	Through the multi‐modal approach, the ACCP service has developed a process to acquire advanced airway management skills including endotracheal intubation Success Rate contrast favourably with national and international success rates	Level II B	JBI: include (Checklist for prevalence studies)
4	Jackson and Carberry (2014) Scotland	Report of evaluation of the specific activities, workload and patterns of prescribing of advanced nursing practice posts within a critical care setting	Quantitative evaluation study		Training of ANPCCs to match the roles of the trainee doctor in critical care with only a few tasks transferred to the consultant on duty Addition of ANPCCs to unit efficiency by taking over responsibility for and monitoring of national data input Utilisation of ANPCCs in any ICU/HDU setting to safely contribute to service provision	Level III C	JBI: include (Checklist for prevalence studies)
5	Kerr and Macaskill (2020) Ireland	Exploration of ANPs (Emergency) perceptions of their role, positionality and professional identity	Qualitative narrative inquiry Bourdieu's concept of habitus, field and capital	*n* = 10 purposive sampling; achieved data saturation	5 main themes: Participants career pathways Personal and professional transitions Role dimensions and core concepts Position within the organisation Emergent professional identity	Level III B	JBI: include (Checklist for qualitative research)
6	Laird et al. (2020) UK	Gain of insights into the future scope and role of future advanced nurse practitioners in stroke care from the perspectives of key stakeholder	Qualitative Interviews Inductive Content Analysis	*n* = 18 purposive sampling	4 four main themes: The lynchpin of the acute stroke service An expert in stroke care Person and family focused Preparation for the role	Level III A	JBI: include (Checklist for qualitative research)
7	Lee et al. (2018) UK	Outlining of practical approach of how the programme was developed and is being delivered at CCU	Discussion	n.a.	Positive experience with implementation of ACCNP model, according to early and enthusiastic buy‐in from key stakeholders, comprehensive job description, ability to use FICM ACCP curriculum and close work relationship between trainee and supervisor New advanced practice role offers opportunities to fill gaps in medical workforce, improving continuity of patient care, provide mentoring and training for less experienced staff as well as offering a rewarding clinical role Successful demonstration of implementation and integration of ACCPs at CCU Implementation is labour intensive and requires significant input from clinicians and academics Success of ACCP implementation is due to a strong partnership between hospital and university, but also buy‐in from key stakeholders	Level V A	JBI: include (Checklist for text and opinion)
8	Naranjo et al. (2023) Colombia	Understanding of social processes experienced by nurses and the meanings underlying autonomy in adult ICUs	Qualitative Study Grounded Theory Approach	*n* = 15	4 categories: Adaption process Applicability of autonomy exercise Building autonomous competence Limitations to the exercise of autonomy	Level III A	JBI: include (Checklist for qualitative research)
9	Solberg et al. (2023) Norway	Identification of required competencies of APNs working with patients in CCUs in Norway	Qualitative Study 4 focus group interviews	*n* = 18	APNs require the following competencies to meet needs of patients: Intrapersonal skills (i.e., self‐awareness, motivation and commitment, strong mental health and upholding ethical standards) Advanced clinical decision‐making skills (i.e., integration of theory and practice, complex practical and technical skills, dealing with increased delegated responsibility and taking the lead in managing increased practice complexity) Interpersonal skills (i.e., peer guidance, practising collaboratively and the ability to position oneself)	Level III A	JBI: include (Checklist for qualitative research)
10	Tuite and George (2010) USA	Discussion of development of Rules of Evidence (ROE) Committee (implementation of evidence‐based practice EBP)	Discussion	n.a.	Successful strategies for the implementation of EBP include support of nursing administration, multidisciplinary approach to involve all experts, nursing faculty collaboration, and shared leadership between nursing and critical care medicine Institutions must remain focused on decreasing cost and increasing quality, CNS is instrumental in ensuring performance of these directives CNS can work to improve quality and ensure that the care provided is based on sound scientific evidence Committee format was very successful in providing an avenue to facilitate the EBP process	Level V B	JBI: include (Checklist for text and opinion)
11	Weatherburn and Greenwood (2023) Australia	Description and explanation of the role of the intensive care nurse within the medical emergency team (MET) to develop an understanding of the intensive care nurse role, the way it is enacted, and their responsibilities within the team	Qualitative study Constructivist Grounded Theory Research Approach	*n* = 12	4 key concepts: Systematic framework for decision‐making Figuring it out Directing care Patient safety	Level III A	JBI: include (Checklist for qualitative research)
12	Webb et al. (2023) Australia	Development of the foundation of the adult ICU NP role in Australia	Article		ICU NP Program: An innovative model of care is described, demonstrating potential benefits for patients and their families Healthcare System Benefits: The program shows promise in supporting advanced practice nursing development in regional and rural Australia Addressing Workforce Issues: The model is recognised for its potential in addressing future workforce issues in the Intensive Care Unit (ICU) Clear Role and Structure: The program establishes a clear role and structure for the integration of Nurse Practitioners (NPs) in adult ICU settings Ongoing Research: Research is underway to evaluate the impact of the NP role within the ICU, indicating a commitment to ongoing improvement and assessment	Level V A	JBI: include (Checklist for text and opinion)
13	Wolf et al. (2017) USA	Identification of skills being performed by APRNs in emergency care settings. Exploration of types of training. Description of competency validation	Exploratory mixed‐methods study Self‐report survey, focus group interviews	*n* = 146 (completed surveys) *n* = 24 APRNs (focus group)	No standardised education in APN Few programs address the need for APRNs Fragmented training, various validation of skills for NPs and CNS The presence of other providers (specifically physicians), institutional culture, and state boards of nursing that regulate practice affect APRN practice autonomy	Level III A	MMAT: include
14	Wolf et al. (2023) USA	Explication of the application of the Emergency Nurses Association core competencies and definition of CNS role in emergency care. Alignment of current CNS practice in emergency settings with the National Association of CNS core competencies	Quantitative Design Exploratory Descriptive Approach	*n* = 285	Valid Competencies: ED‐situated clinical nurse specialists possess valid competencies in frequency and importance Credentialing Gap: Despite this, they are not fully credentialed or practicing to their full education and licence extent Reasons for Gap: Professional, legislative, and environmental factors contribute to these limitations Potential Benefits: Increasing full‐practice clinical nurse specialists could enhance education, clinical quality, and overall system efficacy Role Delineation: Recommendations include clearly defining the role by professional organisations and academic programs, reflecting in institutional job descriptions Communication with Administrators: Findings should be shared with nurse administrators to highlight the value of clinical nurse specialists in patient care, nursing, and organisational advancement Further Research: Additional studies are needed to identify interventions and incentives promoting full practice to the extent of licensure and education	Level III A	JBI: include (Checklist for prevalence studies)

#### 3.3.5 Data Analysis and Synthesis

Data extraction was performed in tabular form by one researcher (VB) and reviewed by the whole research team. To analyse the data, thematic analysis according to Kuckartz and Rädiker (2023) was conducted in MAXQDA to identify recurring themes and patterns related to APN competencies and scope of practice in critical care. Deductively, and to guide the synthesis of data, the core and central competencies of Hamric's model of Advanced Practice Nursing (Tracy et al. 2022) were used as the conceptual framework. These deductive categories were: (1) ethical decision‐making, (2) collaboration, (3) leadership, (4) evidence‐based practice, (5) consultation, (6) guidance and coaching, and (7) direct clinical practice. The researchers also inductively identified the additional category of (8) education and expanded the framework accordingly.

All included studies were coded line‐by‐line. Initially, one‐third of the material was open‐coded to refine the category system, followed by axial coding and integration into the predefined framework. Deductive codes were used to cluster predefined competencies, while inductive coding allowed for new, data‐driven subthemes. Data reduction involved condensing coded segments to identify core categories relevant to critical care APN roles. To compare findings, similarities and differences across countries and healthcare systems were explored.

The combination of an inductive and deductive approach enabled a structured yet flexible synthesis, whereby international variations and recurring patterns in APN competencies were systematically mapped and categorised. The resulting thematic structure forms the analytical foundation of the following results section. The synthesis presented there distinguishes between APN core competencies and scope of practice and is visually summarised in two tables. Table 2 presents the thematic categories and subthemes related to competencies, while Table 3 provides a structured overview of clinical and procedural activities that define the scope of practice across international settings. In this review, scope of practice refers to the set of clinical activities, decision‐making responsibilities, and organisational roles that APNs are authorised or expected to perform in critical care settings. This includes procedural autonomy, interprofessional responsibilities, and context‐specific role enactment shaped by institutional or regulatory frameworks. In contrast, competencies are defined by the knowledge, skills, and attitudes required for the effective fulfilment of roles.

**TABLE 2:** Competencies of APNs in critical care.

Datasynthesis			
Main themes	Subthemes	Description	Sources
Education (*n* = 5)		Development of extended skills relies on the combination of local courses, degree courses and departmental needs Some extended skills are part of standard emergency nurse competencies Frameworks and curricula to guide the experience required for a nationally recognised emergency care In some practical skills, a working knowledge rather than personal competence was appropriate Educational preparation at Master level is essential Various curricula for different patient groups (adults, children), depending on career intentions and scope of practice Developing ACP skills is divided in academic preparation and experiential learning Competency‐based approaches with a shared curriculum valuing unique contributions of different health professionals may lead to richer skill mixes while maintaining quality and safety Development of ACP role in emergency department needs to be accompanied by a structured education programme Absence of existing internationally and nationally recognised frameworks allows individual trusts to make their own arrangements Experience of a learning curve, educationally and practically by undertaking master's degree	Crosbie et al. (2014), Crouch and Brown (2018), Jackson and Carberry (2014), Kerr and Macaskill (2020), Webb et al. (2023)
Ethical decision making (*n* = 1)		Decisions about what should be done and what is right and responsible Critical care APNs protect the integrity of patients and practice according to ethical standards	Solberg et al. (2023)
Collaboration (*n* = 8)		Interprofessional teamwork supports quality of care, resources and time management Creation of specialist nurses improve opportunities to learn safe and evidence‐based methods and have impact on other health professionals Higher effectiveness through shared workload ACPs provide advice and support and can bridge the gap between other health professionals Lack of support by either managers or other health professionals Interprofessional supervision is important to address organisational contexts, power dynamics and role clarity No clear leadership or roles allocedted in medical emergency teams, shared responsibility and teamwork between nurses and doctors If acuity increases, APNs are asked to perform procedures that are not normally part of their daily practice	Crosbie et al. (2014), Crouch and Brown (2018), Kerr and Macaskill (2020), Laird et al. (2020), Solberg et al. (2023), Tuite and George (2010), Wolf et al. (2017), Weatherburn and Greenwood (2023)
Leadership (*n* = 8)	Organization (*n* = 6)	Success of implementation of ACCP requires strong partnership between hospital, university and key stakeholders Responsibility of nurses often given by senior department physician Work of ACCPs depends on hospital administrative systems Different departments have different policies Restrictions of practice development and scope of practice often by immediacy and authority	Crouch and Brown (2018), Denton et al. (2022), Kerr and Macaskill (2020), Laird et al. (2020), Lee et al. (2018), Tuite and George (2010), Wolf et al. (2017), Webb et al. (2023)
	Policy/national trust (*n* = 4)	Nursing practice depends on political and social drivers Changes in organization and professional boundaries have lead to reconstruction of habitus of ANPs Limitations in scope of practice due to restrictions in institution and state boards of nursing Wide variation in scope of practice exists as determined at multiple regulatory levels including state, institutional, and physician group/employer Australian ICU NPs scope of practice is definded by the nurse practitioner standards of practice by the Nursing and Midwifery Board of Australia	
	Costs (*n* = 4)	Reduction of staffing costs through ACPs Cost‐effectiveness issues in terms of implementing NPs	
	Workforce (*n* = 4)	Separation of ACPs from nursing rota Changing demands require flexible, adaptable, and well‐trained workforce Various UK models where ACP contributed substantially to workforce Significant efficiencies in patient through‐put	
Evidence based practice (*n* = 7)		There is some evidence for safety and effectiveness of NPs, however, there is lack of common definition of role, scope of practice and standardisation Portfolios of skills are evidenced with research articles ACPs are encouraged to read articles that are related to particular areas of clinical interest The academic underpinning of the role is essential for establishing the credibility of the role ANPs felt they were underachieving in research due to lack of time or skills Enhanced coordination is pivotal to the delivery of EBP CNS as key factor for facilitating and implementing EBP Decision making based on scientific evidence	Crouch and Brown (2018), Kerr and Macaskill (2020), Laird et al. (2020), Lee et al. (2018), Naranjo et al. (2023), Tuite and George (2010), Wolf et al. (2023)
Consultation (*n* = 5)		CNS position entails regular liaison with staff and patients across different sites CNS position has expertise in a certain area and entails assessment, organisation, and support in various aspects of staff, patients, and their families CNS as contact person in case of emergency	Crosbie et al. (2014), Laird et al. (2020), Wolf et al. (2017), Wolf et al. (2023)
Guidance and coaching (*n* = 10)		Dedicated clinics provide excellent training opportunities to gain higher degree of exposure in a safe environment Development of knowledge and skills in a variety of settings. Clinical supervision and support should be multiprofessional and with the help of protocols An ANP needs to be able to pass on knowledge and expertise to other colleagues NPs influence in terms of empowerment, education, and role modelling APNs in critical care lead by example NPs educate and train medical staff, allied health professionals and nurses CNS develope interventions to reduce work‐related stress	Crosbie et al. (2014), Crouch and Brown (2018), Denton et al. (2022), Jackson and Carberry (2014), Laird et al. (2020), Lee et al. (2018), Solberg et al. (2023), Wolf et al. (2017), Webb et al. (2023), Wolf et al. (2023)
Direct clinical practice (*n* = 13)	Environmental conditions (*n* = 9)	Ineffectiveness through inappropriate patient transfers Assurance of competence through competency‐based portfolios NPs mainly provide service or manage patients with conditions where there was a protocol or guideline Protocols as limit of ability to respond to client individually Shift of workload from doctors to NPs; they regularly undertake hidden work to facilitate safe and effective patient management Compared to non‐teaching hospitals, CNSs working in teaching hospitals spend significantly more time in their CNS role	Crosbie et al. (2014), Crouch and Brown (2018), Denton et al. (2022), Jackson and Carberry (2014), Kerr and Macaskill (2020), Laird et al. (2020), Lee et al. (2018), Naranjo et al. (2023), Solberg et al. (2023) Tuite and George (2010), Weatherburn and Greenwood (2023), Wolf et al. (2017), Wolf et al. (2023)
	Activities/functions (*n* = 6)	Higher level of practice = more invasive skills; high level of autonomy and complex decision‐making. CNS provide and manage care of complex and vulnerable patient groups. Traditional roles and responsibilities are converging; essential to prepare clinicians in terms of patient safety and quality Enhancement of patient experience through ACP role ACP role in emergency department comprises leadership and specialisation in minor injuries Leadership as a core competency Knowledge, confidence and assertiveness as a foundation for leadership, engagement in decisions and advocacy Use of different title for distinction from staff Role is responsive to demands of changes in infrastructure, policy, and local need Involvement in research and academic activities Different levels of practice lead to variation in pay, title and role, and professional and public confusion in function ANP role is essentially clinically based and patient‐focused. NPs as alternative to doctors	

	Knowledge (*n* = 12)	ACCP role enables improving of continuity of patient care, monitoring, training for less experienced staff Knowledge acquisition as a social process CNS role enables the development of respect and trust between CNS and patients, allows better handling of practical and physiological issues Scope of practice may not be the same as the full curriculum described case mix. Expression of concerns regarding skill of staff ACCPs independent patient assessment and action plan allows prompt review, informed decision‐making, prioritisation and discharge planning Competence to authorise blood and blood products as important aspect of ACCP role adding to seamless and consistent care delivery ANP needs to have knowledge of the best practice and to share it Difference to bedside stroke nurse: advanced assessment skills, prescribing and administering lysis regime, autonomous clinical decision‐making, execution of diagnostic, analytical and clinical judgement skills ANP should not dismiss fundamental nursing skills ANPs must possess advanced clinical skills; they must demonstrate a wider scope of practice Standardisation of education, role and scope of practice could reduce need for protocol led care Advanced practice is about the ability to collect required data for good clinical reasoning and deductive logic Combination of hard (clinical emergency) and soft (communication and organisational) skills, as well as clinical and diagnostic reasoning skills	
	Authority/responsibility (*n* = 7)	ACPs are responsible for having knowledge of intensive care medicine and a number of specialist skills It is in the responsibility of the respective nurse to complete and then present the portfolios Prescribing is vital to autonomous practice in ED ANP role added to unit efficiency by taking over responsibility for and monitoring of national data input Disparity between what ANPs should be doing and what was permitted Comprehensive clinical assessment and problem solving as central element of practice	
Communication (*n* = 7)	Nurses want to give more information than only on their own actions Giving information as important element of nurses' responsibilities Effective, patient focused communication to reduce the likelihood of dehumanising care Communicate in complex situations in a culturally sensitive way APNs in critical care need to be self aware of professional limitations and open to criticism		

**TABLE 3:** Scope of practice of APNs in intensive and critical care setting.

Scope of practice	
Skills	Author
Tracheostomy care	Crosbie et al. (2014)
Tracheostomy tube changes	Crosbie et al. (2014), Webb et al. (2023)
Support emergency services	Weatherburn and Greenwood (2023)
Resuscitation	Lee et al. (2018)
Discharge planning	Crosbie et al. (2014)
Weaning and decannulation assessment	Crosbie et al. (2014)
Prescription of weaning and decannulation plans	Webb et al. (2023)
Cannulation	Crouch and Brown (2018)
Insertion of central venous catheters (PICCs, CVCs, dialysis catheters)	Webb et al. (2023)
Insertion of arterial catheters	Webb et al. (2023)
Request (order) diagnostic tests/scans (X‐rays, blood, CTs, MR)	Jackson and Carberry (2014), Laird et al. (2020)
Pain management	Jackson and Carberry (2014)
Manage critically ill patients	Lee et al. (2018)
Coordinate care	Lee et al. (2018)
Primary case coordination	Webb et al. (2023)
Handling ventilator treatment	Solberg et al. (2023)
Change ventilator mode or setting within defined limits	Solberg et al. (2023)
Clinical patient assessment	Jackson and Carberry (2014), Kerr and Macaskill (2020), Laird et al. (2020)
Handling extracorporeal membrane oxygenation machines (ECMO)	Solberg et al. (2023)
Patient examination (physical)	Jackson and Carberry (2014)
Formulate differential diagnoses	Wolf et al. (2023)
Prescribing	Jackson and Carberry (2014), Laird et al. (2020)
Documentation	Jackson and Carberry (2014)
Discharge	Kerr and Macaskill (2020)
Use of Optiflow without medical order	Solberg et al. (2023)
Lysis administration	Laird et al. (2020)
Administer GTN Infusion (treatment of acute left ventricular failure)	Laird et al. (2020)
Assessment via National Institute of Health Stroke Scale	Laird et al. (2020)
Management of acute stroke	Laird et al. (2020)
Prescription and administration of medicines (e.g., anaesthetics, analgesic, neuromuscular blocking agents, vasopressors)	Webb et al. (2023)
Prescribing antimicrobials	Jackson and Carberry (2014)
Administer oxygen therapy	Weatherburn and Greenwood (2023)
Administer IV fluids	Weatherburn and Greenwood (2023), Webb et al. (2023)
Prescribing blood products	Jackson and Carberry (2014)
Prescribing fluids	Jackson and Carberry (2014)
Prescribing electrolytes	Jackson and Carberry (2014)
Prescribing opioids	Jackson and Carberry (2014)
Pain killers	Fawdon and Adams (2013)
Prescribing unlicensed medication	Jackson and Carberry (2014)
Discontinue drugs	Jackson and Carberry (2014)
Change drugs/doses	Jackson and Carberry (2014)
Order/administer analgesia	Weatherburn and Greenwood (2023)
Advanced physiological monitoring	Lee et al. (2018), Solberg et al. (2023), Weatherburn and Greenwood (2023)
Advanced organ support	Lee et al. (2018)
Airway assessment	Denton et al. (2022), Webb et al. (2023)
Advanced airway management	Denton et al. (2022)
Endotracheal intubation	Denton et al. (2022)
Endotracheal intubation of paediatric patients	Denton et al. (2022)
Bag valve mask ventilation	Denton et al. (2022)
Laryngeal mask airway	Denton et al. (2022)
Provide airway support	Weatherburn and Greenwood (2023)
Advanced life support	Denton et al. (2022)
Develop/implement process change	Tuite and George (2010)
Create education tools	Tuite and George (2010)
Educate staff on practice change	Tuite and George (2010)
Transfer of intubated patients	Webb et al. (2023)
Decision‐making and leadership in ICU	Naranjo et al. (2023)
Developing autonomy in ICU nurses	Naranjo et al. (2023)
Teamwork and autonomy consolidation	Naranjo et al. (2023)

#### 3.3.6 Presentation of Findings

Findings were synthesised and presented according to the thematic categories derived from Hamric's model, ensuring alignment with the conceptual framework. Tables and figures, including a PRISMA flowchart, were used to visualise the search process and summarise the key results. The following section presents the results of the thematic synthesis structured according to the conceptual framework outlined above.

## 4 Results

In this section, the findings of the integrative review are presented. Based on Hamric's model, the results are categorised into competencies and scope of practice of APNs in critical care settings. The synthesis draws on both deductive coding according to the conceptual framework and inductive analysis to capture subthemes emerging across the included studies. This approach allows for the integration of diverse international perspectives while identifying consistent thematic patterns.

The research team included 14 international publications. These were conducted in the UK (*n* = 6), Australia (*n* = 2), the USA (*n* = 3), Ireland (*n* = 1), Colombia (*n* = 1), and Norway (*n* = 1). Of these, two and six publications had a quantitative and qualitative design, and one had a mixed‐methods design. Further, the researchers included one short communication, one symposium, two discussion papers, and one original research article (Webb et al. 2023).

### 4.1 Competencies

APNs in critical care have a multitude of competencies that enable them to provide quality care. Such skills are important in enabling the APNs to address the complexities of critical care. The following sections discuss the identified competencies and how they affect patient outcomes and the healthcare system.

#### 4.1.1 Ethical Decision‐Making

APNs in critical care are very much involved in ethical decision‐making, especially in areas involving the management of issues such as end‐of‐life care, patient autonomy, and rights versus clinical outcomes. According to Kerr and Macaskill (2020), ethical frameworks guide them through such difficult situations.

#### 4.1.2 Collaboration

Another core competency for the APNs in critical care settings is collaboration, whereby they act as the main communicator within healthcare teams. These findings align with earlier reports highlighting the lack of clearly defined leadership roles and responsibilities in medical emergency teams, which reinforces the need for shared accountability between nurses and physicians (Tuite and George 2010). Crosbie et al. (2014) note that APNs ensure effective interprofessional teamwork and smooth coordination of staff. Laird et al. (2020) point out that APNs enhance outcomes, acting as the linchpin within the critical care teams. Kerr and Macaskill (2020) report on how the APNs manipulate the relationships to maintain the team dynamics and clarify the role. According to Weatherburn and Greenwood (2023) APNs become members of the medical emergency teams and through this contribute toward collaborative efforts.

#### 4.1.3 Leadership

Leadership roles include overseeing clinical operations and resource planning. According to Jackson and Carberry (2014), it falls within the scope of a manager to oversee the flow of patients and staffing, ensuring the running of the unit. Crouch and Brown (2018) emanate that APNs contribute to policy formulation and leadership for the bettering of the delivery of care. Particularly, the APN leadership has emphasised the management and development of strategic frameworks of care, as indicated by Denton et al. (2022), for improving patient outcomes.

#### 4.1.4 Evidence‐Based Practice

APNs play an important role in instituting evidence‐based practices in critical care. According to Crouch and Brown (2018), APNs lead research and guideline integration into practice to ensure that patient care meets the best standards. Jackson and Carberry (2014) also indicate that APNs critically review evidence to develop and enhance protocols of care. In particular, Lee et al. (2018) found that APNs were central in facilitating evidence‐based interventions while acting as mediators between disciplines, which supports the integrative function of APNs in clinical decision‐making.

#### 4.1.5 Consultation

In many instances, APNs are responsible as a consultant to healthcare teams. This is usually the provision of expert advice on problematic cases. Crosbie et al. (2014) highlight that APNs advise the nursing staff and the physicians on most matters relating to patient management strategies. Kerr and Macaskill (2020) state that APNs are advisory roles in strained clinical settings while Tuite and George (2010) discuss the consultation role of APNs for enabling evidence‐based practice.

#### 4.1.6 Guidance and Coaching

APNs are role models within the critical care environment, contributing to the junior personnel and interdisciplinary team development. Jackson and Carberry (2014) talk about APNs being used in some practices to manage staff and coach them to further develop their clinical skills. In addition, APNs play a key role in coaching and mentoring staff through structured clinical supervision, as shown in a study by Wolf et al. (2017), which emphasises their contribution to workforce development and stress reduction. Laird et al. (2020) cite leading competency development training programmes for the staff, ensuring quality in care. This outlines the role of APNs in this area concerning preceptorship, advanced peer guidance, and dealing with students at the master's level. Furthermore, structured training models led by APNs, such as those reported by Weatherburn and Greenwood (2023), provide safe learning environments that support the acquisition of advanced competencies through mentoring. Wolf et al. (2023) describe the role as that of mentoring and further discuss it within critical care environments in the United States.

#### 4.1.7 Direct Clinical Practice

APNs are highly involved in critical care, performing advanced and intricate clinical practices. Standardised clinical portfolios have been described as useful tools to assure competence across settings, particularly in high‐acuity environments (Wolf et al. 2023). Jackson and Carberry (2014) explain that the role of APNs in critical care includes intubation and management of ventilators. According to Denton et al. (2022), APNs autonomously assess their patients and make prompt decisions by considering symptoms of sudden deteriorations.

#### 4.1.8 Education

Education is considered as being the vehicle for developing advanced competencies of APNs. Crouch and Brown (2018) forward competency‐based education, while Laird et al. (2020) believe it is mentoring by APNs themselves that develops competencies within teams. Weatherburn and Greenwood (2023) add weight to this view by emphasising that the training programmes should combine theory and practice. An overview of the results of all categories is given in Table 2.

While the previous section focused on the core competencies exhibited by APNs in critical care, the following section outlines the specific scope of practice observed in the reviewed studies, including clinical procedures, autonomy, and interprofessional roles.

### 4.2 Scope of Practice

While the previous section focused on the core competencies of APNs in critical care, the following section outlines the scope of practice—that is, the range of roles, responsibilities, and clinical activities APNs are authorised or expected to perform. This includes their degree of autonomy, the procedures they carry out independently, their involvement in interprofessional care, and how these roles are shaped by institutional, national, and regulatory frameworks. The thematic synthesis highlights international variation as well as common patterns in how APNs are integrated into critical care settings.

These findings reflect a broad and evolving scope of practice internationally, encompassing not only clinical expertise but also advanced decision‐making and collaborative functions. A structured summary of these role components, as identified across the included studies, is presented in Table 2.

#### 4.2.1 Advanced Clinical Knowledge

APNs in critical care perform advanced clinical procedures typically reserved for physicians, such as advanced airway management (e.g., endotracheal intubation, larynx mask placement, tracheostomy care, and tube changes) (Denton et al. 2022; Crosbie et al. 2014; Webb et al. 2023) and management of ventilator settings (Solberg et al. 2023). They also handle cannulation and central venous catheter insertion (Crouch and Brown 2018; Webb et al. 2023). APNs manage critically ill patients requiring advanced organ support, such as ECMO and ventilator therapies, and provide emergency interventions, including stroke lysis administration (Lee et al. 2018; Solberg et al. 2023; Laird et al. 2020). Their role also includes decision‐making and leadership within multidisciplinary teams, fostering autonomy and confidence in dynamic critical care environments (Naranjo et al. 2023).

Education and mentorship are central to the APN role. APNs lead training programmes, mentor junior staff, and implement evidence‐based practice changes to enhance team capacity and professional development (Tuite and George 2010; Laird et al. 2020). Continuous integration of theoretical knowledge with practical skills strengthens APNs' autonomy and competency, as emphasised by Naranjo et al. (2023). APNs involvement in evidence‐based practice fosters a culture of continuous improvement and excellence in care delivery. Naranjo et al. (2023) emphasise that such leadership roles require APNs to navigate complex barriers, including institutional regulations and societal perceptions, to effectively advocate for patient care and nursing autonomy.

#### 4.2.2 Autonomous Decision‐Making

APNs are also important in formulating differential diagnoses and making diagnoses based on patient presentation. According to Wolf et al. (2023), APNs have the autonomy to order pharmacologic interventions, including analgesics and vasopressors (Webb et al. 2023), and to manage and discontinue medications and adjust doses (Jackson and Carberry 2014). This level of autonomy is necessary for timely and effective patient care in high‐acuity settings (Jackson and Carberry 2014; Webb et al. 2023). However, as Naranjo et al. (2023) point out, achieving autonomy is often a gradual social process influenced by institutional and social factors. For example, nurses' autonomy can be hindered by protocol‐based interventions and institutionally imposed restrictions, highlighting the need for a supportive environment to foster independence. As the authors emphasise, though, autonomy in nursing is deeply rooted in continuous professional growth, ethical practice, and overcoming both personal and systemic barriers to ensure holistic, quality care.

#### 4.2.3 Collaborative Approach

APNs play a vital role in critical care settings, combining advanced clinical skills, leadership, and education to address critical workforce challenges and foster a culture of excellence. They significantly contribute to care coordination by facilitating transitions between care settings, managing patient discharge planning, and leading multidisciplinary teams to ensure comprehensive care delivery and optimise patient outcomes (Lee et al. 2018; Crosbie et al. 2014; Weatherburn and Greenwood 2023; Webb et al. 2023). Additionally, APNs mentor and train junior staff, drive evidence‐based practice, and implement quality improvement initiatives, further solidifying their indispensability in critical care worldwide (Lee et al. 2018).

The wide scope of practice underlines how important APNs contribute to the high demands of critical care settings. Further, advanced competencies enhance patient outcomes and contribute to efficient and effective communication in healthcare delivery systems globally.

Such combined roles in Austrian critical care practice could therefore be enormously rewarding, especially in bridging gaps in workforce capacity and respective expertise. These results highlight a complex and diverse set of competencies and roles that APNs fulfil internationally in critical care. The implications of these findings, as well as their relevance for the Austrian context, are discussed in the following section.

## 5 Discussion

This discussion is structured according to the two key aspects addressed in the research questions: APN competencies and scope of practice in critical care. The interpretation of findings is guided by Hamric's model of advanced practice nursing (Tracy et al. 2022), which served as the conceptual framework for data synthesis. This model emphasises the integration of advanced clinical knowledge, ethical responsibility, collaboration, evidence‐based practice, and leadership as central to advanced nursing roles. By linking the thematic findings to this framework, the discussion highlights international commonalities and challenges and reflects on implications for the Austrian healthcare system, particularly regarding educational structures, role implementation, and legal boundaries.

### 5.1 Competencies—Ethical Decision‐Making

There is good international support for the importance of ethical decision‐making by APNs and, in particular, in critical care settings. Tracy et al. (2022) relate the review findings to note that APNs also have a dual role as clinicians and patient advocates with often complex situations in relation to ethics, such as end‐of‐life care and informed consent. However, ethical training and clarity of the role for nurses in Austria are still limited (Glarcher and Lex 2020). Wheeler et al. (2022) pointed out that structured ethical training programmes are necessary, which could be a model when developing a framework in Austria with the intention of supporting APNs in terms of dignity and autonomy of the patients.

### 5.2 Competencies—Collaboration

While collaboration is the cornerstone of APN practice, it still differs a lot in every healthcare system. For instance, in the UK and Australia, APNs are part of multidisciplinary teams that design care pathways to reduce treatment delay (Hassmiller and Pulcini 2020). In the Austrian healthcare system, one sees little evidence of an identifiable pathway for integrating APNs into team‐based care. In this regard, the gap in support for interprofessional collaboration needs structural changes involving shared decision‐making models and team leadership roles of APNs. With great potential, APNs lead the management of care delivery, resources, and staff globally. According to Rafferty et al. (2019), when APNs are empowered to work as clinical managers, efficiency and patient satisfaction improve. Austria, however, has a very strict hierarchical system within healthcare settings, mostly leaving little room for such roles to be applied to APNs.

### 5.3 Competencies—Evidence‐Based Practice

The integration of evidence‐based practices is another core competency of APNs, with global models demonstrating their effectiveness in applying research to clinical protocols. Melnyk and Fineout‐Overholt (2022) emphasise that APNs' leadership in evidence‐based practice improves patient outcomes and reduces healthcare costs. However, Austria faces barriers, including the lack of career paths, the recognition of any regulatory or government body, and clearly defined roles (Pirhofer et al. 2022).

### 5.4 Competencies—Education

The foundation of APN competencies lies in education. Structured master's‐level programmes have an important role, and the evidence from around the world confirms this fact. As the ICN (2020) explains, the nurses from countries where there are well‐developed programmes for APN training more often report higher clinical skills and job satisfaction. The lack of professional educational pathways is considered one of the biggest obstacles facing critical care APNs in Austria. Further, APN skills and job profiles depend on the commitment and organisational opportunities of individual health care executives (Glarcher and Lex 2020).

### 5.5 Scope of Practice—Advanced Clinical Knowledge

Advanced clinical capabilities such as airway management, central line insertions, and ventilator adjustments are core features of the APN role in critical care. For example, U.S. studies, such as that by Wolf et al. (2023), demonstrate how APNs performing these activities reduce physician workload and enhance team efficiency. However, in Austria, restrictive regulations prevent nurses from assuming these responsibilities (Health and Nursing Act 1997b, §20). Such policy reforms that widen the scope of APN practice could address workforce shortages, particularly in areas where health disparities are great.

### 5.6 Patient Outcomes

Numerous international studies have demonstrated the positive impact of APNs on clinical outcomes, patient satisfaction, and healthcare efficiency. For example, Melnyk and Fineout‐Overholt (2022) reported significant improvements in evidence‐based practice implementation and cost reduction in APN‐led teams. Similarly, Rafferty et al. (2019) found that empowering APNs in clinical leadership roles improved care coordination and staff satisfaction. These findings support the importance of formal recognition and structured implementation of APN roles, particularly in high‐acuity settings such as intensive care. However, such outcomes have yet to be systematically evaluated in the Austrian context.

While the findings of this review provide valuable insights, several limitations should be considered when interpreting the results.

## 6 Limitations

This review is subject to several limitations. First, the inclusion of studies was limited to English and German publications due to the authors' language competencies. This might have led to the exclusion of relevant evidence published in other languages. Second, the search period was restricted to the past 16 years, and despite expert input and librarian support, relevant studies may have been missed due to variability in terminology or indexing. Third, the included literature varied widely in terms of country of origin, stages of APN role development, and methodological design. This heterogeneity limits the generalisability of the findings.

However, this variability also reflects the international diversity of APN practice and was consistent with the integrative aim of the review. To strengthen methodological rigour despite these limitations, the research team adhered to the integrative review framework by Whittemore and Knafl (2005), applied systematic data extraction, and conducted qualitative content analysis according to Kuckartz and Rädiker (2023). These steps ensured transparency and analytical consistency. Nonetheless, the findings should be interpreted with caution, particularly in light of contextual differences between healthcare systems.

Despite these limitations, the review contributes meaningfully to the international discourse on advanced nursing roles in critical care and offers orientation for future research and policy development in Austria.

## 7 Conclusion

Against this backdrop of limitations and insights, this chapter presents the main conclusions and directions for policymaking, educational development, and further inquiry in the Austrian context. APNs demonstrate a comprehensive set of competencies internationally, which enables them to provide high‐quality care and address critical healthcare challenges effectively. Their role is pivotal in enhancing patient outcomes, fostering interprofessional collaboration, and advancing healthcare systems. This review highlights the transformative potential of APNs, particularly in critical care settings, where their contributions can optimise care delivery and improve efficiency.

The findings emphasise the importance of expanding the scope of practice for APNs within Austria. Regulatory and policy reforms are essential to empower APNs to take on more autonomous roles, contributing to the sustainability of the healthcare system. Lessons from countries with established APN roles underscore the benefits of integrating advanced practice nursing into critical care, including improved patient outcomes and reduced system strain.

Education and training remain central to realising the full potential of APNs. Developing specialised programmes that align with international standards would strengthen the role in Austria and ensure readiness for complex healthcare environments. Collaboration and shared decision‐making models further enhance their ability to lead initiatives and streamline care pathways.

By implementing targeted reforms and fostering a supportive environment, Austria can unlock the potential of APNs, ensuring a resilient and high‐quality healthcare system that meets the demands of modern care.

## 8 Implications

The findings highlight a significant gap between the international competencies and scope of practice of APNs in critical care and the current situation in Austria. Addressing these disparities requires comprehensive reforms, including the establishment of standardised master's‐level education programmes and clear regulatory frameworks to support the development of APN roles. These measures would not only enable Austrian APNs to meet international standards but also enhance their ability to address complex healthcare challenges, particularly in critical care. Furthermore, fostering a supportive environment for APNs, including opportunities for interprofessional collaboration and leadership, is essential to unlocking their full potential. By bridging the gap between international standards and national practices, Austria could improve the quality and efficiency of its healthcare system and better meet the demands of critical care environments.

## Author Contributions

Made substantial contributions to conception and design, or acquisition of data, or analysis and interpretation of data; V.‐K.B., K.P., R.E.‐M. Involved in drafting the manuscript or revising it critically for important intellectual content; V.‐K.B., K.P., R.E.‐M., M.G., A.E. Given final approval of the version to be published. Each author should have participated sufficiently in the work to take public responsibility for appropriate portions of the content; V.‐K.B., K.P., R.E.‐M., M.G., A.E. Agreed to be accountable for all aspects of the work in ensuring that questions related to the accuracy or integrity of any part of the work are appropriately investigated and resolved; V.‐K.B., K.P.

## Conflicts of Interest

The authors declare no conflicts of interest.

## Supporting information


**Data S1:** jan70179‐sup‐0001‐Supinfo.docx.

## Data Availability

The data that support the findings of this study are available from the corresponding author upon reasonable request.
