# Supplementary material for: International Competencies of Advanced Practice Nurses in Critical Care: An Integrative Review
Source: J Adv Nurs. 2025 Sep 29;82(5):4766–85. doi: 10.1111/jan.70179 (PMC13069244; doi:10.1111/jan.70179)
Supplement: Supplementary file 1 — Data S1: jan70179‐sup‐0001‐Supinfo.docx. [file JAN-82-4766-s001.docx]

Supplement 1: Search String PubMed

(("nurse practitioner*"[All Fields] OR "nurse practitioner*"[MeSH Terms] OR "advanced critical care practitioner*"[All Fields] OR "clinical nurse specialist*"[All Fields] OR "nurse clinician*"[MeSH Terms] OR "nurse clinician*"[All Fields] OR "critical care nurse*"[All Fields] OR "critical care practitioner*"[All Fields]) AND ("practice patterns, nurses"[MeSH Terms] OR "practice patter*"[All Fields] OR "knowledge base*"[MeSH Terms] OR "knowledge base*"[All Fields] OR "critical care nursing"[MeSH Terms] OR "critical care nursing"[All Fields] OR "scope of practice"[MeSH Terms] OR "scope of practice"[All Fields] OR ("scope"[All Fields] OR "scopes"[All Fields] OR "scoping"[All Fields]) OR "advanced practice nursing"[MeSH Terms] OR "advanced practice nursing"[All Fields] OR "nurse s role*"[MeSH Terms] OR "nurse s role*"[All Fields] OR "role*"[All Fields] OR "professional competenc*"[MeSH Terms] OR "professional competenc*"[All Fields] OR "skill*"[All Fields]) AND ("intensive care unit*"[All Fields] OR "intensive care unit*"[MeSH Terms] OR "emergency medical service*"[MeSH Terms] OR "emergency medical service*"[All Fields] OR "intermediate care facilit*"[MeSH Terms] OR "intermediate care facilit*"[All Fields] OR "critical care unit*"[All Fields] OR "intensive treatment unit*"[All Fields])) AND ((humans[Filter]) AND (english[Filter] OR german[Filter]) AND (2007:2023[pdat]))
